# Credibility Contests: Media Debates on Do-It-Yourself Coronavirus Responses and the Role of Citizens in Health Crises

**DOI:** 10.3389/fsoc.2020.592666

**Published:** 2020-11-04

**Authors:** Sonja Erikainen, Ellen Stewart

**Affiliations:** Centre for Biomedicine, Self and Society, Usher Institute, University of Edinburgh, Edinburgh, United Kingdom

**Keywords:** DIY, COVID-19 responses, citizen science, news discourses, credibility contests, boundary work

## Abstract

During the early months of the COVID-19 pandemic in Europe and North America, news outlets ran a series of stories reporting on “do-it-yourself” (DIY) coronavirus responses that were created and implemented by citizens. This news discourse exemplifies and can illuminate wider shifts in the roles of citizens in science, as individuals outside professional science institutions are becoming more actively involved in scientific knowledge production than before, while the epistemic authority of professional “expert” scientists has been increasingly contested. This paper focuses on DIY citizenship, taking news discourses on citizens' DIY coronavirus responses as a lens to explore wider questions around the changing ways in which the roles of different public health actors are delineated and represented under conditions of significant social and epistemic uncertainty. We aim to shed new light on the nature of—and the role of the news media in mediating—the credibility contests and boundary work that is currently at play around DIY citizenship. We do so by focusing on four discourses: polarized discourses around DIY face masks and hand sanitisers; delineation of credible from incredible interventions and actors around DIY coronavirus treatments and tests; delineation of professional science from “fringe” citizen science; and discourses declaring that “we're all in this together.” We conclude that making sense of these discourses requires a thorough appreciation of the context in which they emerged. Our analysis reveals how emancipatory accounts of DIY citizenship can mask structural inequalities underlying who can and is expected to “do-it-themselves,” and how.

## Introduction

During the early months of the COVID-19 pandemic in Europe and North America, several news outlets ran stories reporting how citizens were responding and contributing to pandemic mitigation. Many especially highlighted so-called do-it-yourself (DIY) coronavirus responses put forward by individuals and communities. In the UK, for example, a local newspaper reported that “government ministers and MPs … expect the biggest health care crisis in a generation to be managed as a DIY project by the British public” (Wiltshire Times, 2020), while the *New York Times* (NYT) noted that “D.I.Y. coronavirus solutions are gaining stream” across the US too: “while government officials scramble to find a solution, do-it-yourself makers are pressing ahead” (NYT, 2020a).

These reports were part of a wider news media discourse on DIY coronavirus responses that surfaced as the pandemic begun to disrupt healthcare systems, economies, and citizens' lives, while government-directed pandemic responses not only varied across different countries, but were also perceived by many as inadequate (see Hale et al., 2020; Tanne et al., 2020). The emergence of a discourse on DIY coronavirus responses exemplifies wider shifts in the articulations of citizenship, the roles of citizens in science, and public health science in particular: citizens are increasingly regarded as agents as well as subjects of science, with active roles in, for example, citizen science, DIY science and public involvement in science, while concurrently, the epistemic authority of professional scientific and public health “experts” has been increasingly contested (Erikainen et al., 2019). These shifts came to light in a pronounced way during the early months of the COVID-19 pandemic as citizens took action in conditions of uncertainty.

News media discourses on DIY coronavirus responses can illuminate the changing framing of citizens' roles and knowledge claims. In this paper, we use discourse analysis to map how news outlets framed and evaluated citizens' DIY coronavirus responses during the early months of the pandemic in Europe and North America, focusing especially on the UK and US contexts. Building on Gieryn's (1999) conceptualization of “credibility contests” and “boundary work” around science with respect to public health science,^1^ we map how these DIY responses were positioned and evaluated in relation to the knowledge claims of conventional public health authorities. We aim to shed new light both on the nature of the credibility contests and boundary work that is currently at play around citizen participation in science, and how the news media mediates these contests and boundary work. In particular, we consider how credibility and epistemic authority over public health was constructed under conditions of an unprecedented public health crisis and uncertainty of a global pandemic.

In what follows, we first unpack the changing roles of citizens in science and innovation, including the contemporary movement and manifestations of DIY citizenship and volunteerism. We then consider key discourses evident within our analysis of news media. We conclude that news media discourses on DIY coronavirus responses shed light on wider debates around citizen participation in science in the current socio-economic and socio-technological context. Making proper sense of news discourses themselves requires a thorough appreciation of the context in which they emerged. Furthermore, engaging with news discourse reveals how emancipatory accounts and mobilisations of the DIY notion may mask structural inequalities that underlie who can and is expected to “do it themselves,” and how.

### DIY Citizenship, Boundary Work, and Credibility Contests in the Digital Era

Citizen responses to the coronavirus pandemic can be conceptualized in light of what Ratto and Boler (2014) among others have termed “DIY citizenship,” which denotes an emergent frame of often politically-interventionist practices at the intersection of tensions between experts and novices, individuals and communities, and politics performed by governments and DIY grassroots democracy. DIY coronavirus responses during the early months of the COVID-19 pandemic resonate with the wider contemporary DIY- and “maker” movements, where people are engaged in doing and making all manner of things from electronics to genetic engineering to furniture to sewing themselves (see Davies, 2017). Despite varying in both character and scope, Wehr (2012) argues that these movements collectively represent a move toward independence and self-reliance, away from reliance on experts, professionals and larger governance and capitalist systems. Those engaged in DIY practices generally share the idea that “the tasks that many are ready and willing to have others do for them can (and perhaps should) be done by one's self” (Wehr, 2012, p. xi). Indeed, DIY can be positioned as a solution to experiences of loss of control in the contemporary context; people aiming to take control over their daily lives when wider social, scientific, economic and political forces feel beyond it (Wehr, 2012). The “DIY citizens” notion invites us to consider, not only how and when citizens participate in the social and political construction of selves, worlds, and environments, but also how they do so in ways that challenge conventional power divisions, understandings of scientific or political activity, and normative understandings of how things should be done (Ratto and Boler, 2014).

DIY citizenship is importantly connected with volunteerism, a separable but overlapping form of social and political activity, which emphasizes “collaboration and service over challenge and opposition” (Greer et al., 2014). While there has been considerable debate over what constitutes “volunteering” and who should be considered a “volunteer” (see Cnaan et al., 1996), literature on volunteerism increasingly recognizes “informal” and “spontaneous” alongside more organized volunteering, especially during crises (e.g., Whittaker et al., 2015). This includes not only individual volunteering responses to crisis response but also “emergent organizations” formed during or immediately after an emergency has occurred “when needs are not being met, or it is perceived that needs are not being met, by other organizations” (Whittaker et al., 2015, p. 360). Both DIY citizenship and these forms of volunteering suggest that there is a need, as Stewart (2016) has argued, to expand our accounts of citizen and public participation beyond the “formal” and “invited.”

DIY citizenship and informal volunteering are shaped by wider socio-economic contexts where citizenship discourses and practices have undergone problematic shifts. Governments across the world have increasingly advanced neoliberal modes of governmentality involving not only state rollback and public service privatization but also corresponding political discourses encouraging citizens to be active, entrepreneurial, and responsible for their own lives and health (Drake, 2011). As Drake (2011) has argued, neoliberal governmentality techniques result in offloading responsibilities, including over health and well-being, from the state to individuals and local communities, while citizens are pushed toward modes of citizenship that cultivate self-governance. Citizens' “empowering” control over their own health may easily translate into amplified expectations or even obligations to maintain and promote one's health (i.e., do-it-yourself) rather than relying on public healthcare services. The DIY notion in these discourses is conjured as a means of greater self-determination and control for citizens, but the reality can be that unpaid volunteers are recruited to substitute public sector health workers (Chidgey, 2014). Neoliberal governmentality has been accompanied with calls for groups of local volunteers and non-profit organizations to take up roles left open by public service funding cuts (Bloom and Kilgore, 2003; Brown and Baker, 2012). This displacement of state responsibilities onto individuals and communities is not experienced evenly, but is refracted through existing societal structural inequalities, including gendered traditions of labor, and racialised and classed community experiences of state services (Dean, 2015).

Changing citizen innovation practices in crises should be understood also within the wider context of the so-called digital era (Erikainen et al., 2019), which has allowed greater access to scientific knowledge online, and for people to circulate and produce it with less reliance on professional scientists' and experts' mediation. In the healthcare sphere, digital technologies have facilitated developments like participatory medicine and citizen science, where individuals without professional scientific training or credentials are increasingly involved in the production of scientific knowledge and innovation especially through digital means (Erikainen et al., 2019). Unprecedented access to and circulation of scientific knowledge in the digital era has resulted in concerns over the proliferation of misinformation and “fake news” in the context of a wider crisis of social authority and public mistrust (Marres, 2018; Bratich, 2020). It has even been argued that digitally mediated social life is characterized by contested politics of truth, where, to borrow Michael Gove's words, “people have had enough of experts” (see Marres, 2018).

Citizens' increasingly active roles in healthcare and direct engagement in the production of health-related knowledge (including via DIY science that goes beyond institutionally-legitimated modes of scientific research and practice) might suggest health professionals' and “experts”' traditional authority in delimiting the boundaries of knowledge is becoming outdated. Yet, Dommett and Pearce (2019) have highlighted that media coverage of “expertise” and “experts” has increased in recent years and, rather than being side-lined in favor of citizens' voices, experts are being summoned to arbitrate a wide range of subjects. Concurrently, there is insufficient empirical evidence to support any strong claims about how publics more generally perceive experts, including what role and importance is assigned to certified expert knowledge (Dommett and Pearce, 2019). Indeed, Brante (1993, p. 181) has argued that actually, in areas and during times characterized by scientific controversy, “experts are the primary actors” in social debates. In the popular media context and wider public discourses, experts are still principally positioned as having privileged knowledge, and are given key roles in facilitating and often directing public debate, societal progress, and decision-making (Dommett and Pearce, 2019).

Rather than assess an overall gain or loss for expert authority, we mobilize the idea of credibility contests to explore discourses of “expert” and “DIY” responses to the coronavirus. Through his original framework of “boundary work,” Gieryn (1999, p. 2) argued that “newspapers … and cyberspace are fat with credibility contests,” whereby “experts bearing science are enlisted everywhere to defend all sides and all opinions with putatively objective, reliable, and accurate facts.” The notion of “credibility contests” is central to Gieryn's (1999) conceptualization of “boundary work” around science, which takes place when the nature and content of “real science” is discursively demarcated from various categories of science “posers” and “fringe science,” such as “pseudoscience” or bad science but also, importantly, amateur science. Mainstream science is often pitted against the fringe, where “fringe science” can be understood to capture a range of heterogenous activities at the outskirts of institutionally legitimated science, forming a liminal sphere at the edges of “science proper” (Vaage, 2016). Such fringe endeavors may aim at “shifting the current ideas of who is entitled to conduct research in the life sciences, and how such research should be done” (Vaage, 2016, p. 127). Credibility contests, in turn, involve different actors and players who articulate and enact knowledge claims about the “true” nature of reality, claiming their knowledge as epistemically authoritative, while often relegating contradictory claims as untrustworthy or inaccurate (Gieryn, 1999). This tends to take place via appeals to science that reproduce its epistemic authority, even while what exactly “science” amounts to, or the truths that it contains, may be framed differently by different parties in the contests (Gieryn, 1999).

Especially in fast moving areas of scientific and public debate like the COVID-19 pandemic, news outlets easily become central mediators of credibility contests, and a part of the making of “science” as a cultural sphere (Gieryn, 1999). Especially under conditions of significant scientific uncertainty and disagreement like the early months of the COVID-19 pandemic where evidence was only emerging and “experts” lacked consensus, the mass media become engaged in interpreting and disseminating public health messages to the larger population. The boundary work that the media mediate and undertake is performative; it not only conceptually delineates credible information but, in so doing, also directs which kinds of public health interventions come to be considered legitimate and are promoted, made available, and taken up, while others become side-lined. In relation to DIY coronavirus responses, news outlets became directly engaged in boundary work and credibility contests concerning both citizens' role in health crises, and disputes between different “experts” who disagreed with each other and made different knowledge claims (Martin et al., 2020a). They mediated these contests in a context where people were not only faced with multiple and discrepant claims that were located in different epistemic spaces (Gieryn, 1999) but also in a context where citizens are increasingly asked to take active roles in managing health and carrying responsibility over it, but in ways delimited by pre-existing expert-led ideas about which kinds of citizen activities are (and are not) useful.

## Materials and Methods

This paper reports a discourse analysis of news media coverage of DIY coronavirus responses in Europe and North America in February and March 2020, focusing especially on the US and UK contexts, and on how the concept of DIY was used as a framing tool for media depictions of citizen responses to the pandemic. These two regions were chosen because they are similar enough to enable direct meaningful comparison between the regions, but nonetheless sufficiently different to enable consideration of how the discourses around DIY coronavirus responses were shaped by the wider socio-political contexts where they manifested. It is notable that the contemporary media landscape is a hybrid of traditional and digital communication forms. A range of technologies mediate communication with the effect that news coverage is constructed and received in a fluid manner, as people increasingly rely on digital news sources and use social media to access news (Haw, 2020). Yet, the popular news media continue to be a key social source of information about scientific, medical and health-related developments, and while it would be inaccurate to frame the relationship between news media depictions and public perceptions as causally deterministic, the news media nonetheless influence public knowledge and attitudes (Hiebert and Gibbons, 2000).

**Figure 1:**
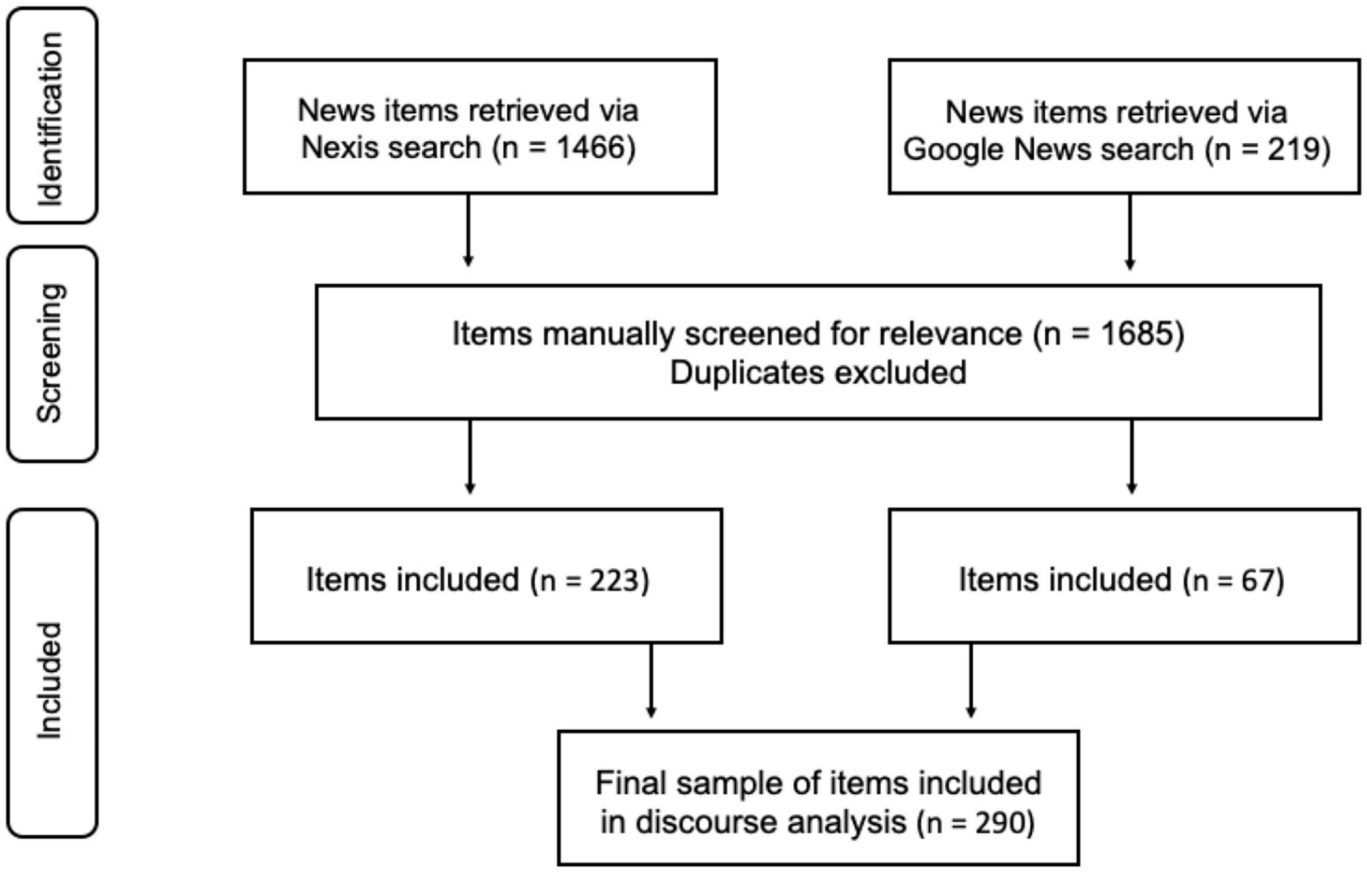
Data collection process (modified from Moher et al., 2009).

We used the Nexis newspaper database to retrieve relevant news items, supplemented with a Google News search for further items that may have been excluded from Nexis. We used the key words “DIY” and “do-it-yourself” combined with different spellings of COVID-19 to search the databases. The search was regionally confined to North America and Europe, limited to news published in English, and temporally restricted to the period between 1st of February and 31st of March 2020. The Nexis key word search retrieved 1,466 items and Google News retrieved 219 items. All were then manually reviewed for relevance, which was determined simply on the grounds that the news items had to cover content about coronavirus DIY responses. 223 items from Nexis and further 67 items from Google News were included into the final dataset after excluding duplicates, resulting in 290 items in total (see Figure 1).

The news items were disproportionately focused on the US and UK, reflecting in part the inclusion of only English language publications. 156 items were published by US-based news outlets while 72 were UK-based. Most outlets had some level of international circulation, however, with some being very international, making it somewhat difficult to accurately represent regional divisions in distribution. Many items covered multiple kinds of DIY activity concurrently, making it difficult to numerically reflect the prominence of sub-topics, but 80 were principally focused on DIY face masks, 40 on DIY hand sanitisers, 36 on the maker movement and related citizen-led innovation, 22 on DIY coronavirus testing, and 14 on DIY coronavirus treatments and alternative medicine. These form the sections of our analysis below. The rest primarily covered health-related DIY developments generally, discussed several of the above topics relatively equally, or were principally focused on DIY lifestyle (e.g., beauty, home improvement, gardening) during the pandemic lockdown. Here we focus on health-related developments.

The data was analyzed using Parker's (1992) discourse analysis framework, where “discourse” can be understood as ways of constituting knowledge through representational practice. Parker's (1992) framework involves asking what objects and subjects are referred to and how they are positioned, while mapping a picture of the world the discourse presents and its relationship to other discourses. It involves identifying the institutions that are reinforced or subverted by the use of different discourses, and looking at ideological effects and power relations; who gains, who loses, which perspectives are voiced, and which are side-lined. Focusing on news items as the texts or “occasions” through which discourses are realized, applying Parker's framework involved the following process: all news items included in the study were subjected to multiple rounds of close reading. During initial rounds, the key topics of fucus were identified, and the items were grouped by topic. During subsequent rounds, for each item, information was derived and recorded about how the concept of DIY and other concepts like expertise were discursively produced and framed in the news item; how subject positions like the citizen, amateur, expert or specialist were designated; and what wider discourses and discursive contexts foregrounded these framings/designations. Then, similarities and differences across the discursive framings/designations embedded in the individual news items were derived and interpreted to produce an account of the four key discursive frames that we analyse below.

As this study was temporally limited to February and March 2020, regionally confined to North America and Europe, and included only content published in English, it should be taken as a “snapshot” of media discourses in specific contexts. As such, it cannot offer a comprehensive account of the discourses on DIY coronavirus responses as they developed over time, across the globe, or even across Europe where much news coverage takes place in languages other than English. Especially, it cannot speak to the complexities involved as these discourses stretch beyond European and North American contexts, including low-/middle-income countries characterized by dissimilar forms of inter- and intra-national disparity in public health access, resources and capacity. Moreover, as this study is focused on news media discourses, it has very limited capacity to illuminate discourses taking place in other public spheres, including social media.

## Results

### Polarized Discourses: DIY Face Masks and Hand Sanitisers

In March and February 2020, news outlets across Europe and North America reported that the healthcare sector was facing significant shortages of personal and protective equipment (PPE) due to an unpredicted spike in demand, while commercial retailers both offline and online were running out of stocks of face masks and hand sanitisers. These two commodities became totemic within debates about DIY responses to the pandemic. The UK Mail Online (2020a) reported that “frontline NHS doctors are being forced to buy face masks from DIY stores because of nationwide shortages amid the coronavirus crisis,” while The Scotsman (2020) added that social workers were resorting to making “DIY virus kits for home visit protection.” The *Minnesota Public Radio* declared, moreover, that “for some medical providers, P-P-E is being spelled D-I-Y” (MPR News, 2020).

While public debates emerged concerning the question of whether people should be wearing face masks in public in general (see Martin et al., 2020b), there was a sub-debate waged in the media that concerned the question of whether people should be making and wearing DIY masks in particular. Several news outlets reported that some hospitals and individual healthcare professionals had called for local communities to make hand-made masks as a last resort to mitigate the PPE shortage: St. Paul Pioneer Press (2020) for example noted that “scores of people” are “answering pleas from hospitals, doctors and nurses so desperate for personal protective equipment amid the viral pandemic that they've turned to the public, saying do-it-yourself face masks are better than nothing.” Contextualized by such reports, news outlets ran stories about “volunteers [who] sew masks in their homes in bid to help healthcare workers” (Mail Online, 2020b), showing what the Business Insider (2020) called “an underground economy” of people making masks. Many others distributed DIY face mask designs and advice for making masks both for frontline workers and personal use.

As these stories were surfacing, however, many news outlets begun publicizing an explicitly contrasting message, warning people against DIY mask-making. Most of these messages quoted or explicitly built on advice from various medical and public health “experts” and “specialists,” constructing a discourse that positioned DIY face masks as ineffective or outright dangerous. These messages included headlines like “why DIY masks won't keep you safe from COVID-19” (WebNews, 2020) and “please do not make a DIY surgical mask during the Coronavirus outbreak. Allow a medical doctor to explain” (Men's Health, 2020). The CBS News (2020a) quoted an “infectious disease specialist” calling DIY mask-making an “online fad” that “may actually do more harm than good.” Indeed, the Mail Online (2020c) reported that while “desperate” people unable to buy face masks due to the shortage have used “carved out melons, plastic bottles, even bras, sanitary towels and lettuce leaves” to cover their faces, the Public Health England head of emerging infections and zoonoses said that when it comes to masks, “I don't think they do any good … physically they are not a prevention.”

While reports like the above initially surpassed in volume those that advocated DIY mask-making as a productive endeavor, toward the end of March, warnings against homemade masks were increasingly challenged by headlines stating that, “actually, some officials say, you should be wearing a homemade mask amid coronavirus pandemic” (Philadelphia Inquirer, 2020). Many of these aimed explicitly to counter claims against DIY mask-making and often did so by building on “expert” or “specialist” advice that directly opposed the warnings against DIY masks. The Washington Post (2020) for example ran a commentary with the headline, “simple DIY masks could help flatten the curve. We should all wear them in public.” It highlighted that there exists “significant scientific evidence” against the idea that homemade masks “are NOT effective in preventing general public from catching Coronavirus,” and stated that “there are good reasons to believe DIY masks would help a lot” (The Washington Post, 2020). Some rather polemical commentaries also emerged, with a NYT (2020b) opinion piece stating that “the top-down conversation around masks has become a case study in how not to communicate with the public,” including because “of course masks work—maybe not perfectly and not all to the same degree, but they provide some protection.” This pro-homemade masks discourse also coincided (and individual news items sometimes cross-referenced) news about government policies in countries like the Czech Republic and Austria that made wearing masks compulsory in public places: Euro News (2020a) reported, for example, that “Czechs … have dug out their sewing kits in recent days to take part in a DIY drive to create their own protective face garbs, after a regulation … made it compulsory to wear either a surgical face mask or other mouth and nose-covering apparel when in public.”

Such polarized discourses were not limited to DIY masks but emerged also in relation to DIY hand sanitisers as a response to the hand sanitiser shortage. Firstly, several news outlets released and re-distributed recipes for making hand sanitisers, many of which cited “expert” advice. The Express Online (2020a,b) advised readers “how to make your own hand sanitiser at home with 3 ingredients” and “how to make hand sanitiser with wine—expert reveals all about DIY gel,” while The Independent (2020) instructed “how to make your own hand sanitiser, according to experts.” Like with DIY face masks, these reports were explicitly countered by opposing messages stating that “experts have warned against making your own DIY hand sanitisers” (The Sun, 2020a) and “do not make hand sanitiser using DIY guides, experts warn” (Sky News, 2020a).

These “expert warnings” principally focused on the fact that many people were reported to be using consumer alcohol products and especially vodka to make hand sanitisers, raising concerns over insufficient alcohol percentages. The Express (2020) news item titled “key reason DIY hand sanitiser NOT enough to kill deadly coronavirus—WARNING,” for example, stated that while “vodka is being suggested as the alcoholic component to kill the virus with homemade hand gels,” “the drinking spirit only has 40 percent of alcohol, which is not enough to kill germs.” It added that “experts warn … that it's easy to mistake the concentration of alcohol to other substances … these recipes are not all easy to follow and could actually cause more harm than good” (Express Online, 2020c). Warnings against DIY hand sanitisers often referred to the homemade substances as “concoctions” and especially underlined concerns about the spread of recipes on social media. CBS News (2020b), for example, highlighted how “experts [are] alarmed as … recipes for homemade hand sanitizer concoctions have been multiplying online,” as many “are turning to social media for information about how to make their own and some of that information is very misleading.” The Marketwatch (2020) added that “some misinformed individuals have … been looking into making sanitizers with liquor … and taking their queries (and dubious success stories) to Google … and Twitter.” In these kinds of reports, citizens were generally positioned as uninformed or even reckless, to the extent that, it was implied, they might take dangerous kinds of self-initiative.

The above discourses illustrate how news outlets were actively engaged, not only in disseminating emerging health information to wider audiences, but also in deciding, almost in real time, upon the value and credibility of the emerging information. They did this centrally by mobilizing the notions of “expert” and “specialist” as indicators of credibility, and, in so doing, reinforced the epistemic authority of “expertise” possessed by professional scientists and public health professionals. The “experts” in question were referenced in ways that supported and lent credibility to different, contradictory truth claims. Indeed, while knowledge concerning the merits and disadvantages of wearing masks in general and DIY masks in particular was only emerging and partial, “experts” were enlisted to provide public health recommendations despite the limited evidence base and lack of scientific consensus (see Martin et al., 2020b). Whatever claims were made about DIY masks and hand sanitisers, however, news outlets centrally relied on “expert” advice and “scientific” evidence, even while the content of that evidence and advice was contested.

The polarized nature of these discourses also illustrates how the role of citizens in managing the COVID-19 pandemic was delineated during its early months. While news items supporting DIY face mask- and hand sanitiser-making efforts generally framed citizens as competent and capable actors in pandemic mitigation, the oppositional discourse that warned against such efforts generally positioned citizens as un-/misinformed actors whose DIY efforts were unlikely to help and, rather, do more harm than good. In this way, the polarized discourses amounted to credibility contests both around the truth value of different knowledge claims, and the competence of different actors to utilize and take action in relation to these knowledge claims.

### Delineating Credible Medical Interventions: DIY Treatments and DIY Testing

Discourses also emerged around DIY treatments and, albeit less evenly, DIY coronavirus direct-to-consumer testing kits. Firstly, several news outlets reported on the proliferation of DIY treatments that were associated with alternative medicine and generally rendered ineffective or outright dangerous in ways that invoked a boundary between credible or “proper” medicine and pseudoscience. Secondly, a related discourse emerged around commercially available DIY home testing kits that were generally rendered fraudulent in opposition to presumed trustworthy government interventions, in way that constructed an association between commercial health products, fraudulent practices, and alternative medicine. In both cases, those who provided and used DIY treatments and tests were generally positioned as misinformed, reckless, or dangerous.

Exemplary of the first discourse is a news item published by the international business-focuses newsletter Quartz (2020):

misinformation about the coronavirus has flooded social media … A widely circulated post … claims you can check if you have the virus by holding your breath for more than 10 s. The information has been shared tens of thousands of times around the world, and it is not true … Ignore “remedies” and “cures.” They don't exist. … We've seen an alternative health publication offering free energy healing, and a Facebook post about shoving colloidal silver up your nose. While neither of those are probably intentional disinformation, they could be harmful if someone sick turns to false cures over actual medical advice.

Many news outlets published items explicitly focused on issuing warnings against scams and myths about the coronavirus, including claims like “cocaine can kill the coronavirus” and “Drinking ‘Miracle Mineral Solution’ can kill the virus” (Telegraph, 2020), with the Huffington Post (2020), among others, stating that “instead of wasting your time and money on scammy products or bad advice, listen to the experts.”

Some news outlets conveyed that “reports of serious injury and even death have begun to surface as people raid their cabinets in search of dangerous ingredients to concoct homemade anti-coronavirus “remedies”' (Fox News, 2020). Among the more widely circulated news stories were reports that “in Arizona, one man died and his wife landed in critical condition after the pair reportedly drank fish tank cleaner allegedly believing that they were ingesting the same anti-malarial medication currently being touted by officials as a possible COVID-19 treatment;” namely, hydroxychloroquine, which is an ingredient in many fish tank cleaning products (Fox News, 2020). Smaller news outlets like the Slash Gear (2020) technology news website also picked this up, with the headline: “public warned against DIY COVID-19 treatments after man dies.” Indeed, in a news item covering the attempt by this couple “to self-treat,” the Targeted News Service (2020), which provides converge of the Washington Bureau, advised that the Association of American Physicians and Surgeons had issued a release stating that “it is risky to talk about what might work for an illness, because people might try to self-treat in a dangerous way.”

These reports were not only directly engaged in defining alternative medicine interventions as scam products, myths, misinformation, and bad advice in opposition to presumable credible “expert advice,” but they also positioned those engaged in using such alternative strategies as misinformed individuals who are endangering themselves in an attempt to create DIY treatments. Reports about the Arizonan couple functioned centrally as a cautionary tale in relation to a wider discourse warning citizens against treatments not provided or approved by “experts.”

Debates around DIY testing delineated credible and trustworthy actors from untrustworthy or fraudulent actors by associating the former with “proper” medicine and the latter with alternative medicine. Several news outlets published reports about commercially available DIY home testing kits that could be ordered online. The Sun (2020b), for example, reported that in the UK, “more than 2,000 people have paid £375 each for a home kit produced by a Harley Street clinic after being refused testing by the NHS.” These can be seen as a part of the wider direct-to-consumer (DTC) medical testing market, which functions principally online, enabling consumers to bypass public healthcare services and information channels to order medical tests that can be self-administered at home, and then sent back to the businesses for analysis (see e.g., Saukko et al., 2010; Curnutte and Testa, 2012). Reports on COVID-19 DIY testing kits generally framed the commercial companies selling these kits as untrustworthy, and the tests themselves as a negative development that should be guarded against. Several news outlets reported that the US Food and Drug Administration (FDA) had “cautioned the public against buying coronavirus disease 2019 (COVID-19) testing kits as these do-it-yourself tools have ‘very low reliability and accuracy.’ COVID-19 testing kits sold online are not approved by the FDA” (ABS-CBN News, 2020). The FDA (2020) itself released a statement that “alerts consumers about unauthorized fraudulent COVID-19 test kits,” advising that the FDA “is actively and aggressively monitoring the market for any firms marketing products with fraudulent coronavirus (COVID-19) diagnostic, prevention and treatment claims.” In stating that it “will take appropriate action to protect consumers from bad actors who take advantage of a crisis to deceive the public by marketing tests that pose risks to patient health” (FDA, 2020), the FDA did not differentiate between different kinds of non-FDA-approved interventions. This was a form of boundary work to define FDA-approved interventions as credible “proper” medicine, while non-approved interventions (i.e., commercial products, alternative treatments used by citizens) were positioned as fraudulent alternative medicine. Irrespective of whether they were DTC DIY swab tests analyzed in commercial laboratories by private sector professionals, or wholly unevidenced products like colloidal silver, they were all positioned as fraudulent and untrustworthy.

By contrast, many news outlets also covered stories about government-directed initiatives to introduce DIY home testing which positioned the proposed tests principally as a positive development, framing the government as a credible and trustworthy actor. In the UK, the Scottish Express (2020) reported on the Public Health England plans to introduce antibody “home tests that can show whether someone has had coronavirus,” adding that the prospect brings “fresh hope for worried NHS staff.” While news items sometimes referred to the importance of ensuring safety and reliability of these tests, they did not question the trustworthiness of the government. Indeed, the main concern expressed around these tests was delay in their distribution. The Mail Online (2020d), for example, reported that the “government was accused of allowing thousands of infected people to walk the streets undiagnosed,” and highlighted that the DIY test rollout “could take MONTHS.”

It is noteworthy that despite differences between national healthcare systems and coverage, there was very little cross-Atlantic difference between the discourses around DIY testing and treatments. Irrespective of region, news outlets generally associated DIY treatments and commercial DIY testing with alternative medicine, untrustworthiness, or fraudulence while framing those providing and using DIY treatments and tests as misinformed or dangerous. By contrast, governments, regulatory agencies, and experts were positioned as trustworthy agents of medicine “proper” that citizens should listen to, instead of attempting to “do it themselves” or rely on presumed fraudulent commercial actors. However, the fact that this boundary work especially in relation to DIY testing kits was done in a context of perceived failure by governments to swiftly launch adequate testing is also noteworthy. This provides explanatory background for why commercial DIY tests and other interventions including alternative DIY treatments may have proliferated; namely, to fill a gap left open by insufficient government response.

### Fringe Science: Citizen Innovation and the Maker Movement

Significant amount of news coverage was focused on efforts by the “maker movement,” including DIY biologists and engineers, to fabricate coronavirus responses. Many news outlets reported that DIY and maker communities were harnessing existing digital platforms and creating new ones to co-produce and disseminate designs of PPE, ventilators and other medical equipment, even a vaccine. With regard to the boundary work around science, DIY science and the maker movement can be understood as examples of “fringe science” (Vaage, 2016).

News coverage of these practices and communities was connected with the above delineation of credible from incredible actors but here, the focus was on discerning the nature and value of amateur science in relation to mainstream science. Indeed, some news outlets reported on the efforts of the so-called DIY biology community to “develop an internationally crowdsourced coronavirus test,” adding that they were “driven by concerns over testing accessibility” (Crosscut, 2020). Crosscut (2020) reported that some makers “are exploring ways to empower their neighbors to take health into their own hands,” tapping into the wider DIY drive toward self-reliance and independence, also quoting one expert stating that professional scientists “have really strict ways to make sure that we don't have false positives and false negatives, and that we have access to real samples … I'm a little bit worried that somebody in their garage might not be able to do all that” (Crosscut, 2020).

Most of this news coverage was, however, focused on citizen scientists' and engineers' efforts to craft and share designs for essential medical equipment such as PPE and ventilators. These reports often emphasized that “hobbyists” and “tinkerers” were organizing themselves via various digital platforms like the Coronavirus Makers Forum that “connects members, extracts insights, and builds bridges to health care institutions and experts” (Forbes, 2020a). Forbes (2020a) reported that in Spain,

thousands of citizens have been connecting online to fight against the shortage of life-saving equipment. From their living rooms and basements, they tinker with ideas and designs, share them, build prototypes and print them out with 3-D printers.

It was highlighted that these communities include “everyone—engineers, students, dads, moms, daughters, sons, people who work in companies, teachers, carpenters. … everyone is contributing and doing what they can to mitigate the crisis” (Forbes, 2020a).

News outlets regularly emphasized that the key motivator for these initiatives was local and global equipment shortage, and the fact that the standard medical innovation process for designing, manufacturing, and gaining regulatory approval for new products was too time consuming and restrictive in the pandemic context. Quoting a member of the local maker movement, the Pittsburgh Post-Gazette (2020) highlighted that in the time it takes a local hospital “to figure out whether they want [DIY face shield], we have individual doctors placing orders for hundreds of these shields” and “they're telling me that they have nothing left.” Similarly, the Euro News (2020b) noted that,

as countries grapple with a shortage of stock, throngs of engineers and 3D-printing enthusiasts have banded together to come up with their own solutions. It comes after doctors have taken to social media to plead for equipment that is in short supply … While much of the supplies that have been created are unregulated, these innovators are working under a single premise: that, in a crisis, alternatives are better than nothing.

Some coverage considered the DIY innovations to be efforts by “home hobbyists” that “amounted to jerry-rigged … devices,” like ventilators that “can't sense how much oxygen is getting into a patient's lungs and aren't nuanced enough to support people with coronavirus caused respiratory distress” (Motherboard, 2020). In reporting how ventilator shortages had “prompted various individuals and groups, for better or worse, to look at MacGyvering their own airway support equipment,” The Register (2020) added that “non-approved devices may present significant safety risks.” Quoting the director of a hospital critical care unit, The Register (2020) noted that while the director considered that “DIY ventilator projects could help in some situations,” they “raise a number of concerns” and “would be difficult to implement in practice based on the principles of safe and effective mechanical ventilation.”

There was a notable amount of coverage on a single initiative by a group of DIY biologists to manufacture a COVID-19 vaccine. Sky News (2020b), for example, reported that a “self-described ‘collective of biohackers’ calling itself CoroHope” are “attempting to crowdfund their own DIY vaccine for the coronavirus,” doing so “completely outside of academia and without the involvement of either the pharmaceuticals industry or government regulators.” While this initiative received coverage in mainstream news outlets, it was especially discussed by alterative outlets such as the *CoinDesk*, a news website focusing on cryptocurrencies like bitcoin. According to CoinDesk (2020), the group is crowdsourcing bitcoin donations to fund their work, and “drawing on bitcoin's decentralized ethos for inspiration,” “bypassing academia, pharmaceutical companies and the … FDA.” Quoting a representative of the group, CoinDesk (2020) conveyed that “cryptocurrency is uniquely able to help with this problem because, like us, it's outside the traditional system … frankly we're not interested in waiting for regulations to try to do good work.” Much of the news coverage around CoroHope in both mainstream and alterative news positioned the group in opposition to traditional biomedical innovation pathways and governance structures, highlighting the constraints that institutionalized structures place around medical innovation “from the fringe” of traditional science infrastructures.

These discourses around DIY science and the maker movement highlight the hybrid space in which citizen innovation is located and show the importance of including maker spaces as a key part of our accounts of citizen and public participation (Pallett et al., 2019). The initiatives picked up by news outlets ranged from fringe efforts that were explicitly located outside mainstream scientific, regulatory and financial systems, to efforts that largely supported or became integrated within mainstream innovation structures. To borrow Light's words (2014, p. 266), these practices are “at once productive and resistant to dominant modes of production; involving innovation in a shape that is unsupported by traditional forms of economic infrastructure” while “drawing on lay insights and potentially crossing multiple categories of societal organization.”

The value and potential of citizen DIY scientists and the maker movement were discursively framed in complex ways in relation to mainstream science and its oversight mechanisms. While many news outlets directed attention to the potentially ineffective or unsafe, experimental and unapproved nature of the DIY products and processes, they generally did not position “fringe” actors as fraudulent or untrustworthy (e.g., in the way that commercial companies producing DIY coronavirus tests were positioned). Instead, citizen scientists were often framed as sitting ambiguously either at the boundaries or outside of professional science, engaged in potentially useful, if risky, activities to supplement or address insufficient pandemic preparedness on part of governments, professional scientists, and other mainstream institutional actors. The role and value of these fringe initiatives remained, in other words, ambivalent.

### “We're All in This Together”: Mass Volunteerism and Nostalgia

As well as the substantive discussions of masks, sanitiser, testing and fringe science, a central, overarching thematic was the “we're all in this together” discourse. This framed DIY coronavirus responses and citizens' role during the pandemic in terms of volunteerism and mass mobilization during great need and uncertainty. The phrase “we're all in this together” was regularly repeated and widely disseminated as a positive message capable of harnessing a collective spirit. Indeed, the Belfast Telegraph Online (2020) commented, not only that the “we're all in this together” message is “over and over again … being drummed in,” but also that “if there is any small solace right now it's that the vast majority of people recognize that and have responded magnificently … you'll see plenty of examples of kindness and caring. Decent people doing their wee bit.” The American business magazine Forbes (2020b) added:

a significant movement, perhaps even a revolution of epic noble intentions, is underway in hackerspaces, makerspaces, and sewing groups to come together and solve a problem to save lives at risk with the Coronavirus. … People of all ages and walks of life are diving in to make a difference. … It is possible that the government and manufacturers will ramp up in a wartime-like effort, but the reinforcement is more likely to come from the people. … Makers, hackers, craftspeople are awesome. Coronavirus does not stand a chance.

This discourse framed citizens as key actors in pandemic mitigation, often mobilizing ‘Homefront’ war analogies. Some news reports focused on the wider re-emergence of a “DIY lifestyle” (e.g., vegetable gardening) during the pandemic, and a potent comparison was made between the WWII Homefront, framing DIY lifestyle changes as a nostalgic return to wartime national unity. As Ginn (2012) has argued, the wartime “dig for victory” discourse and Homefront imaginary particularly in the UK and US continue to resonate because they celebrate wartime as a period where national publics came together. The “we are all in this together” discourse harnesses these wartime imaginaries and nostalgia to construct citizen DIY and volunteerism during COVID-19 in terms of national unity as well as duty. The NYT (2020c), for example, ran an article about “Corona Victory Gardens” arguing that activities like gardening may even be a civic duty. “Gardens flourished on the home front because people were eager to build their own community-based food security, and to cultivate something beautiful and useful in times of great stress and uncertainty” (NYT, 2020c).

Wartime discourses were also mobilized by some news outlets in relation to the mass mobilization of volunteers making face masks. The NYT (2020d) noted that “legions of sewers” had been “called to duty in a matter of days via social media and word-of-mouth,” and they were “building supply chains, organizing workers, managing distribution networks” and “making masks for America, much as a previous generation manufactured ammunition and tended ‘victory gardens’ during World War II.” Wartime imaginaries framed volunteering discourses also in the UK, especially around the NHS and Health Secretary calls for the British public to volunteer to help the NHS respond to the pandemic. The Daily Mail (2020) among others noted that “the response from big-hearted British citizens” was “displaying unvarnished Blitz spirit, these community-minded citizens are pulling together in the national good.”

In harnessing wartime nostalgia, notions of collective empowerment and togetherness, and in celebrating volunteerism and mass mobilization, the “we're all in this together” discourse framed citizens and publics as playing a direct and central role in pandemic response, and reinforced the message that individuals and communities should, and may have a duty to demonstrate good citizenship. Celebratory accounts around citizen mobilization functioned, however, to mask complexities and structural inequities. Firstly, they mask how much of the volunteerism and DIY responses were motivated by perceived insufficient government response to the pandemic and failures of public healthcare infrastructures to protect citizens. As we noted above in relation to DIY treatments and DIY testing, and citizen innovation and the maker movement, much of the DIY citizenship exhibited in response the COVID-19 pandemic was connected to perceived gaps in government responses. Yet, the “we're all in this together” discourse in news items during the early months of the pandemic did not explicitly acknowledge or consider this.

Secondly, they mask structural inequities within the voluntarist movement itself. In an article listing “how you can help with the PPE shortage while staying home,” the Cosmopolitan (2020) addressed those with access to 3D printers, time, skills and capacity required to sew masks and pre-prepare healthy meals for healthcare workers. Forbes (2020c), in declaring that “you are needed,” highlighted that volunteers “skilled in technology, data, design, and operations” including “coding,” “supply chain logistics, and public health, specifically epidemiology” and those able to donate money and equipment were sourced. There was also a notable underlying gendered dynamic to the DIY mask-making movement news discourse that was not explicitly articulated but often seeped through implicitly. For example, the Morning Call (2020) noted that this was, indeed, “a group of women with sewing machines on the ready.” Despite its celebratory tone of togetherness and collective action, the “we're all in this together” discourse was, itself, structured by underlying inequalities that shape whose efforts are valued, celebrated, and, indeed, expected.

## Discussion and Conclusion

The coronavirus pandemic created conditions of significant social and epistemic uncertainty, in which the news media had a heightened role in undertaking boundary work and mediating credibility contests. Our aim in this paper has been to take news media discourses around DIY coronavirus responses during the early months of the pandemic in Europe and North America as a lens to explore wider questions around the changing ways which the roles of different public health actors are delineated and represented in wider discourses. Using discourse analysis focused especially on the US and UK contexts and citizens' DIY contributions to public health during the pandemic, we considered how credibility over public health responses was constructed by news outlets during a time of widespread disagreement over what should be done, why, when, and by whom.

Our analysis suggests that the news media actively worked to delineate credibility and assign epistemic authority to some knowledge claims over others in a situation where relevant scientific and public health knowledge was only emerging. To borrow Gieryn's (1999, p. 203) words, in fast moving and dynamic situations like the early months of the COVID-19 pandemic, “the media had to decide—as they crafted their stories—the credibility, originality, and significance” of emerging facts and knowledge claims. Especially in relation to DIY face masks and hand sanitisers, news outlets advanced polarized discourses amounting to credibility contests around the truth value of different knowledge claims and the competence of different actors to act on these claims. Scientific and public health “experts” were generally attributed with epistemic authority and credibility, even while their claims lacked consensus. Relatedly, via discourses around DIY treatments and tests, news outlets engaged in boundary work to differentiate credible “proper” medicine from fraudulent “alternative” medicine. Albeit in more ambivalent terms, news discourses worked also to differentiate DIY citizen science and maker communities from professional scientists and professional science. They generally rendered the latter safe and effective, and the former a risky even if potentially useful “fringe science” sphere. The news media thus undertook boundary work to delineate the sphere of credible science and knowledge from incredible/false information and fringe science, but also worked to demarcate the role that different public health actors including citizens should have in relation to mainstream forms of knowledge production.

Making proper sense of these discourses requires appreciation of the wider socio-economic and techno-scientific context in which they emerged, and which they illuminate. Many of the DIY responses were implied to have been motivated by perceived insufficiencies in state pandemic responses, including shortages of essential medical and protective equipment, bureaucracy and time delays involved in gaining regulatory approvals for new medical products. DIY responses often arose to address these insufficiencies, including through informal and spontaneous volunteering. In the context of neoliberal modes of governmentality that have proliferated in North America and Europe, and accompanying discourses that have called for individuals to become active citizens responsible for their own health, roles vacated by decreased public funding have been taken up non-professional volunteers. Concurrently, DIY activities are often experienced as a solution to perceived loss of control over individuals' lives, especially during times of great uncertainty like the early months of the COVID-19 pandemic.

Not only did citizens and volunteers take on new roles to respond to the pandemic by “doing it themselves,” but news discourses around DIY coronavirus responses were centrally concerned with making sense of the meaning and implications of these new roles. News outlets took part in and mediated contests over the value of citizen responses and knowledge claims that were made by and about them. While news outlets tended to render the responses of individuals and groups working outside mainstream science and its oversight mechanisms as less credible or problematic, there was nonetheless an underlying and at times contradicting “we're all in this together” discourse advocating DIY pandemic responses as emancipatory ways for “all of us” to do our share. In this sense, there was a tension between news media discourses calling for citizens to contribute and do “things” themselves, and discourses questioning the value of these very contributions to maintain the epistemic authority of governments, professionals, and experts in the face of challenges posed against their authority by commercial actors, non-professionals, and citizens.

Within this contradictory demand, the limitations of emancipatory mobilisations of the DIY notion are doubly demonstrated. The DIY notion is mobilized to support neoliberal governmentality by promoting active “DIY citizenship” as an emancipatory model of agency and self-reliance, at the same time as decreased government funding on essential public services like healthcare means that unpaid volunteers are taking up public sector professionals' roles. However, it additionally intersects with complex, individual circumstances that equip and enable people to contribute differently. Both gendered notions of duty, and (conflicting) professionally segregated notions of valuable skills variegate these nostalgic calls to “doing.” Discourses that declare, or imply, that “we're all in this together” mask structural inequalities that underlie who can and is expected to contribute in different ways, and whose contributions are valued.

## Data Availability Statement

The raw data supporting the conclusions of this article will be made available by the authors, without undue reservation.

## Author Contributions

SE designed the study, undertook data collection and analysis, and was principally responsible for writing the manuscript. ES contributed to data analysis and the theoretical framing and intellectual development of the paper as well as editing the manuscript. All authors contributed to the article and approved the submitted version.

## Conflict of Interest

The authors declare that the research was conducted in the absence of any commercial or financial relationships that could be construed as a potential conflict of interest.

## Footnotes

^1^“Public health” has several meanings. It is, simultaneously, “a scientific field,” “state of health and illness in a population,” and “political programs and infrastructures” aimed at improving population health (Hoeyer et al., 2019, p. 461). We use public health to refer to all of these interconnected dimensions. As Hoeyer et al. (2019, p. 461) argue, the slippage from population health “insights into inequalities” and their determinants into political arguments signals the enduring presence of “biopolitical tension between power and knowledge characteristic of … public health.”
